# Origin of mitochondrial DNA diversity of domestic yaks

**DOI:** 10.1186/1471-2148-6-73

**Published:** 2006-09-22

**Authors:** Songchang Guo, Peter Savolainen, Jianping Su, Qian Zhang, Delin Qi, Jie Zhou, Yang Zhong, Xinquan Zhao, Jianquan Liu

**Affiliations:** 1 Key Laboratory of Qinghai-Tibetan Plateau Ecological Adaptation, Northwest Plateau Institute of Biology, Chinese Academy of Sciences, Xining 810001, Qinghai, China; 2 Department of Biotechnology, Royal Institute of Technology (KTH), 10691 Stockholm, Sweden; 3 Graduate School of the Chinese Academy of Sciences, Beijing 100039, China; 4 Key Laboratory of Arid and Grassland Ecology, College of Life Science, Lanzhou University, Lanzhou 730000, China; 5 Key Laboratory of Biodiversity Science and Ecological Engineering, School of Life Sciences, Fudan University, Shanghai 200433, China

## Abstract

**Background:**

The domestication of plants and animals was extremely important anthropologically. Previous studies have revealed a general tendency for populations of livestock species to include deeply divergent maternal lineages, indicating that they were domesticated in multiple, independent events from genetically discrete wild populations. However, in water buffalo, there are suggestions that a similar deep maternal bifurcation may have originated from a single population. These hypotheses have rarely been rigorously tested because of a lack of sufficient wild samples. To investigate the origin of the domestic yak (*Poephagus grunnies*), we analyzed 637 bp of maternal inherited mtDNA from 13 wild yaks (including eight wild yaks from a small population in west Qinghai) and 250 domesticated yaks from major herding regions.

**Results:**

The domestic yak populations had two deeply divergent phylogenetic groups with a divergence time of > 100,000 yrs BP. We here show that haplotypes clustering with two deeply divergent maternal lineages in domesticated yaks occur in a single, small, wild population. This finding suggests that all domestic yaks are derived from a single wild gene pool. However, there is no clear correlation of the mtDNA phylogenetic clades and the 10 morphological types of sampled yaks indicating that the latter diversified recently. Relatively high diversity was found in Qinghai and Tibet around the current wild distribution, in accordance with previous suggestions that the earliest domestications occurred in this region. Conventional molecular clock estimation led to an unrealistic early dating of the start of the domestication. However, Bayesian estimation of the coalescence time allowing a relaxation of the mutation rate are better in agreement with a domestication during the Holocene as supported by archeological records.

**Conclusion:**

The information gathered here and the previous studies of other animals show that the demographic histories of domestication of livestock species were highly diverse despite the common general feature of deeply divergent maternal lineages. The results further suggest that domestication of local wild prey ungulate animals was a common occurrence during the development of human civilization following the postglacial colonization in different locations of the world, including the high, arid Qinghai-Tibetan Plateau.

## Background

**Figure 1:**
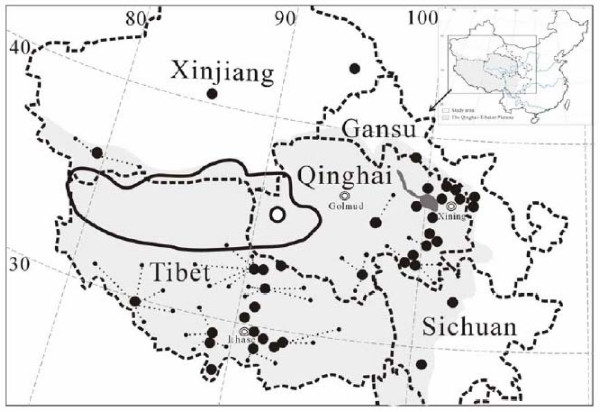
Map showing the geographical distribution of domesticated yaks sampled in five major regions (solid circles, the dotted lines indicate that the yaks concerned were transferred from the areas marked by the small circles to the sampling points marked by the large ones), the current range of the wild yaks (continuous line) and the site (unfilled circle) where skins of eight wild individuals were collected in the Qinghai-Tibetan Plateau (shaded area).

The domestication of plants and animals has played a central role in the socioeconomic transitions from hunter-gatherer organization to agricultural settlement and nomadic pastoralism in human history [1-4]. Domestication, giving rise to organisms with novel combinations of phenotypic traits, was accomplished through human selection for desirable genetic mutations from natural populations. A better understanding of the genetic basis of domestication will help to improve domesticated organisms and open opportunities for new domestications. In addition, genetic evidence has revealed many details, not available through archaeological studies, about the origin and early history of the ungulate domestic meat animals, e.g. sheep [5], cattle [6], pig [7], horse [8], goat [9], dog [10], donkey [11] and water buffalo [12,13]. Elucidating the origin of domesticated yaks (*Poephagus grunnies*) will extend our broad understanding of the main ungulate domestications during the history of human civilization. Domestic yaks are mainly distributed in the Qinghai-Tibetan Plateau (QTP), the largest continuous high elevation ecosystem in the world, occupying nearly 2.5 million km^2 ^of the Asian continent and reaching an average elevation of more than 4000 m a.s.l. (Fig. 1). Both the climate and environments of the Plateau are extremely harsh, its landscapes are barren and the biotic communities are specifically adapted to the extreme conditions [14]. The costs of living here are severe, for example, the nutritional demands and capture costs are very high while the physiological capacity to benefit from successfully procured resources is low [15]. The area is currently occupied primarily by nomadic Tibetan pastoralists [14] and domesticated yaks provide the most important resources (i.e., food, hides, dung fuel and transport power) for them and other populations living here and in adjacent high-altitude regions [14,16,17]. More than 14 million yaks are currently herded in the QTP and the adjacent Asian highlands (North India, Pakistan, Kyrgyzstan, Mongolia and Russia) [14,17]. Morphological data indicate that yaks in areas outside China derived from Chinese yaks [17]. The earliest recorded *Poephagus *fossils originate from the lower Pleistocene [18], which is paralleled by the estimated date, based on molecular data, of the genetic divergence between *Poephagus *and its sister group *Bison bison *around 1.8 million year ago (myr) [19]. It has been speculated that wild yaks were first domesticated in Tibet (Fig. 1) because the earliest evidence of human activity and yak husbandry (Qiang Culture), dating from ca. 10,000 yrs BP, has been found in this region [1]. However, because it is difficult to distinguish between wild and domestic yak remains, this assumed domestication time needs to be confirmed by further evidence. A yak pastoralist society was only well established in the QTP by ca. 4,500 yrs BP [16] and most researchers believe that the yak domestication occurred not earlier than 10,000 yrs BP [17]. Therefore, there is still a lack of independent evidence regarding where and when wild yaks were first domesticated [20]

Previous studies have revealed a general tendency for populations of livestock species to include deeply divergent maternal lineages [5-11,21,22]. Such deep branching suggests that current breeds of some species may have been domesticated in multiple, independent events from genetically discrete wild populations with a wide distribution of wild progenitors [23,24]. However, in some species with a limited distribution of wild populations, e.g., water buffalo, there are suggestions that a deep maternal bifurcation may have originated from a single wild gene pool [12,13]. These hypotheses have rarely been rigorously tested, because of a lack of sufficient wild samples [e.g. for cattle, [6]] and/or phylogeographic structure [e.g. for dogs, [10]] although a multiregional origin has been convincingly shown for pigs by comparing DNA from domesticated pigs and wild boars from various areas around the world [7]. Evidence for this kind of multiregional origin has been obtained for a small domesticated animal, the chicken [22], as well as for the large domestic animals mentioned above. The ca. 15,000 wild yaks that still roam on the plateau [14] (Fig. 1) provide unique opportunities, unavailable for most other domestic animals, to analyze the origins of domesticated yaks and to elucidate their domestication process, especially the development of current maternal lineages.

We present here an investigation of mitochondrial DNA diversity in domestic yaks reared across the QTP. We also surveyed the corresponding DNA variation of 13 wild yaks, eight of which were collected from a single wild population. This maternal sequence is better than other nuclear marker in illustrating domestication events of mammals because it avoids genetic introgression due to hybridization between wild and domestic animals [e.g. [9,10]]. Our main objective in this study was to obtain genetic information on the evolutionary domestication history of modern yaks. The specific goals of this study were to: (1) characterize the maternal diversity of the domestic yaks; (2) examine origins of the main lineages and clades and their correlations with morphological breeds; and (3) explain the probable domestication time of this large animal in the arid QTP based on the genetic calibration obtained in the present study and the available archaeological data.

## Results

### Sequence variation and identified haplotypes

We sequenced mtDNA D-loop region of 250 domestic yaks (see Table 1 for locations) and 13 wild yaks. We also downloaded 25 sequences from GenBank [25]. Using both our data and the published data, 113 variable sites and 87 haplotypes were found in the total sample of 275 domestic yaks and 13 wild yaks. We identified 10 haplotypes in the 13 wild individuals. Only one haplotype was shared by both domestic and wild yaks.

**Table 1:** Sampled breeds and collection information from each province

	Province	Breed	Locality	Number of individuals (downloaded sequence from GenBanK)	Total Number
	Gansu	Unclear	Sunan	8	23
		Tianzhu	Tianzhu	15 (8)	
	Qinghai	Datong	Menyuan	31	125
		Huanhu	Gonghe	3	
		Huanghu	Haiyan	2	
		Huanhu	Gangcha	2	
		Huanhu	Heimahe	2	
		Plateau	Dulan	3	
		Plateau	Yushu	13	
		Plateau	Maqin	32	
		Plateau	Dari	37	
Domestic yaks	Tibet	Jiali	Yanshiping	5	80
		Jiali	Naqu	4	
		Jiali	Anduo	9	
		Jiali	Yangbajing	9	
		Jiali	Dangxiong	9	
		Sibu	Lhasa	1	
		Sibu	Gongga	2	
		Pali	Shigatse	11	
		Pali	Yadong	4	
		Pali	Zedang	10	
		Unclear	Dazi	8	
		Unclear	Zhongba	5	
		Unclear	Unclear	3(3)	
	Sichuan	Maiwa	Hongyuan	5(5)	12
		Jiulong	Jiulong	7(7)	
	Xinjiang	Unclear	Yiwu	10	35
		Unclear	Yecheng	8	
		Bazhou	Hejing	17(2)	
Wild yaks	WYP		West Qinghai	8	13
			Xijinwulan Lake	3	
			unclear	1	
			Kunlun mountains	1	

WYP, the single wild yak population from west Qinghai

### Phylogenetic analyses and networks of the major clades

**Figure 2:**
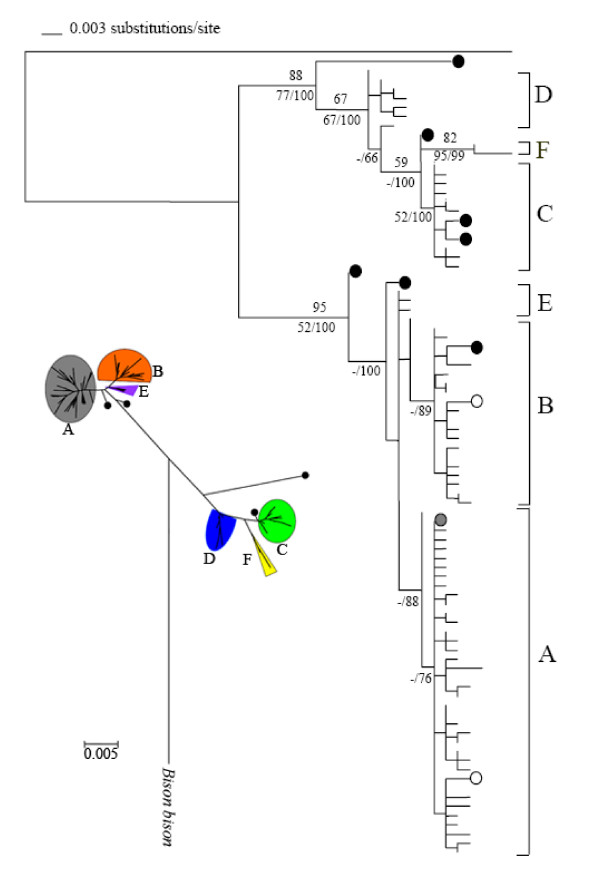
(I). A neighbor-joining (NJ) tree of 87 haplotypes in all yaks constructed by MEGA. The highly divergent mtDNA clades found in domestic yaks were marked with A-G and haplotypes found only in the wild yaks were indicated by circles. (II). Phylogenetic tree of all domesticated (unlabeled) and wild (circles) yak haplotypes constructed by maximum likelihood analysis (-lnL = 1803.55) with the best-fit model (K81uf + I + G), rooted by one sequence of *Bison bison*. The solid circles indicate haplotypes found among the eight wild yaks sampled from a single population in Quinghai and the grey circle indicates the only haplotype found both in the wild population and at high frequency in the domesticated yaks. Numbers below the branches represent support values from maximum parsimony bootstrap and Bayesian inference obtained with the MrBayes program, and numbers above the branches represent the bootstrap values from a fast stepwise-addition search of the maximum-likelihood genetic values obtained with PAUP.

Two deep lineages were identified by the NJ tree (Fig. 2: I) and haplotypes recovered in domestic yaks clustered into six divergent clades (Clades A, B, C, D, E and F). Among them, clade A, B and C contained wild yak haplotypes. This preliminary NJ phylogenetic relationship was further tested by maximum parsimony [see Additional file 1], maximum likelihood analyses and Bayesian analyses (Fig. 1). The aligned dataset for maximum parsimony analyses including all 87 yak haplotypes and one *Bison *sequence contained 637 sites of which 37 were variable but phylogenetically uninformative, and 51 were variable and informative. Parsimony analysis identified 10000 trees with 159 steps, a consistency index (CI) of 0.560, and a retention index (RI) of 0.895. The 50 % majority-rule MP consensus tree (Additional figures: SFig. 1) was basically congruent with the ML tree (-lnL = 1803.55, Fig. 2: II) based on the best fit model (K81 + I + G) although most tentative clades did not receive high bootstrap supports under both ML and MP analyses. Bayesian analysis with a similar topology received moderate to high supports for identified clades A, B, C and F, but failed to support the other clades (D and E). However, all these analyses suggested that 87 haplotypes from both domestic and wild yaks clustered into two well-supported lineages (Fig. 2). This finding is consistent with findings of multiple lineages in most domesticated animals [e.g. [5-9]] as well as previous researches of domestic yaks [25,26]. However, the two lineages we detected contain haplotypes from both domestic and wild yaks. Ten wild yak haplotypes were distributed throughout the total tree. Six of them were found to cluster with Clades A, B and C while the other four are situated at the basal positions of the two lineages.

**Figure 3:**
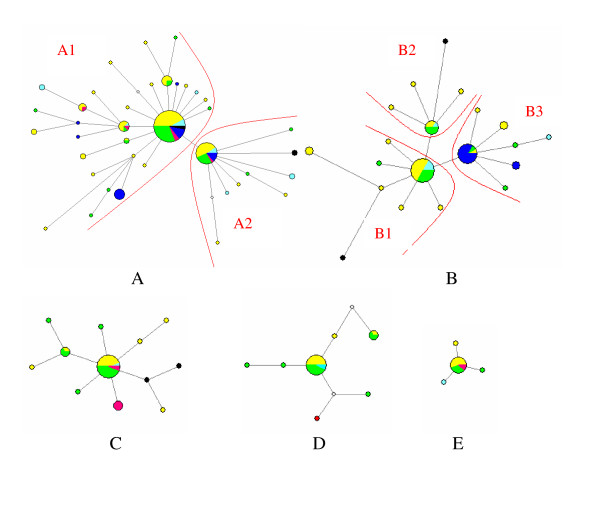
Parsimonious median-joining networks depicting the genetic relationships among mtDNA yak haplotypes of phylogenetic Clades A, B, C, D and E. The sizes of the circles and colored segments are proportional to the haplotype frequencies in the datasets. Colors within circles: black, wild yaks; yellow, Qinghai; green, Tibet; pale-blue, Gansu; red, Sichuan and dark blue, Xinjiang. Red lines indicate subclusters discussed in the text.

Clades C and E, were each dominated by a single high-frequency haplotype clustered with less frequent halotypes (Fig. 3). However, this type of pattern was not found in Clades A, B and D. In each of these cases, two or three major haplotypes, represented in several regions at high frequency, clustered with less frequent haplotypes.

### Geographical distribution of lineages and their correlations with current breeds

Clade A occurred in all regions, Clade B in all regions but Sichuan, and Clade C in all regions but Xinjiang. A total of 56.4% of domestic yaks had haplotypes belonging to Clade A and 87.3% had types belonging to Clades A, B or C. Thus, these three clades constitute a common source for a large proportion of the mtDNA variation in all domestic yaks. Clades D and E were also found in all regions except Xinjiang, but in only 5–10% of the individuals, while clade F was only found in two individuals in Xinjiang. Clades A-E were found in nearly all geographic regions, and at similar frequencies, in all populations (Table 2). The similarity of the genetic variation among regions can be further seen in the phylogenetic networks of clades A-E (Fig. 3). In each of these networks there are a few central haplotypes, found in most geographical regions and at high frequencies, surrounded by less frequent types that are mostly unique to a specific region.

There is also no clear correlation between the mtDNA phylogenetic clades and the 10 morphological types (breeds) of yaks sampled (Table 3), except that haplotypes of the Huanhu breed nested within one of the two main lineages, in Clades A and B. The haplotypes of all other breeds were found to cluster within clades from both of the two deeply divergent lineages. For example, individuals of the Tianzhu breed, which differ from those of the other breeds in that they are white rather than black, nested within Clades A, B, D, and E (Fig. 2). This pattern suggests that mapping of morphology of each breed to the maternal phylogeny is impossible because most clades comprise more than one breed and some haplotypes were shared by all morphological breeds.

**Table 2:** Numbers and proportions of individuals, and numbers of haplotypes and unique haplotypes representing the phylogenetic Clades A, B, C, and D in each of the regions.

Region	N	Nh (U)	Hd	Clade A		Clade B		Clade C		Clade D		Clade E		Clade F	
				
				N (P)	Nh (U)	N (P)	Nh (U)	N (P)	Nh (U)	N (P)	Nh (U)	N (P)	Nh (U)	N (P)	Nh (U)
Qinghai	125	46 (32)	0.93 ± 0.01	74 (59.2)	22 (16)	24 (19.2)	13 (10)	13 (10.4)	6 (4)	8 (6.4)	3 (1)	6 (4.8)	2 (1)	0	0
Tibet	80	30 (16)	0.91 ± 0.02	39 (48.8)	12 (7)	13 (16.3)	6 (3)	13 (16.3)	5 (3)	11 (13.8)	5 (3)	4 (5.0)	2 (1)	0	0
Gansu	23	13 (6)	0.93 ± 0.03	15 (65.2)	7 (4)	5 (21.7)	3 (1)	1 (4.4)	1 (0)	1 (4.4)	1 (0)	1 (4.4)	1 (0)	0	0
Sichuan	12	8 (2)	0.91 ± 0.06	6 (50.0)	4 (0)	0	0	4 (33.3)	2 (1)	1 (8.3)	1 (1)	1 (8.3)	1 (0)	0	0
Xinjiang	35	10 (7)	0.84 ± 0.03	21 (60.0)	6 (4)	12 (34.3)	2 (1)	0	0	0	0	0	0	2 (5.7)	2(2)
Domestic yaks	275	78 (77)	0.92 ± 0.01	155 (56.4)	37	54 (19.6)	18	31 (11.3)	10	21 (7.6)	7	12 (4.4)	4	2(0.7)	2(2)
WYP	8	8 (7)	1.0 ± 0.06	2	1 (0)	1	1 (1)	2	2 (2)	0	0	0	0	0	0
Wild yaks	13	10 (9)	0.96 ± 0.04	4	2 (1)	2	2 (2)	2	2 (2)	0	0	0	0	0	0

N, number of individuals; Nh (U), Number of haplotypes (unique types), Hd, haplotype diversity (Mean ± S.D); N (P), Number of individuals (%) and WYP, a single wild yak population in west Qinghai.

### Genetic diversity and expansion test

As summarized in Table 4, the nucleotide diversities among the clades varied substantially. Generally, Clades C, and E, had relatively low nucleotide diversity and Clade A had the highest diversity. Assuming that domestication of yaks and agropastoralism are likely to have resulted in dramatic, sustained increases in the populations of both the early herders and their herds, genetic indicators of such expansion should be present in the DNA of modern yaks. The star-like phylogeny of three large clades (A, B, and C) is indicative of such rapid expansion following domestications [27]. This demographic expansion hypothesis was further supported by analyses based on two separate procedures. First, each clade produces smooth pairwise mismatch distributions (excluding wild yak haplotypes) that are indicative of expansions [28] (Table 4). Second, Fu's Fs test, which is particularly sensitive to population growth [29], detected significant (*P *< 0.001) signals of departure from neutrality in Clades A, B and C, yielding values of -28.0, -14.2, and -6.6, respectively (*P *< 0.0001).

### Molecular calibration

The coalescent time of two deep divergent lineages was estimated around 131,000 yrs BP (under a constant population size model) or 109,000 yrs BP (under a exponential growth model) when the divergence between yak and *Bison *was assumed to start around 1.8 myr ago under the relaxed molecular clock hypothesis (see Additional file 2). The mean substitution rate was calculated to be 14.6 % per million years or 1 bp substitution per 11,000 years for the analyzed 637-bp region when the constant population size model was assumed. We used this mutation rate to calculate the probable domestication time of yaks. Since the domestications may have resulted in demographic expansion, the initial dates of such events can be estimated using the average mutational distance from the central sequence and the mutation rate [10]. Assuming two origins from wild yak haplotypes for Clade A (the resulting subclusters are indicated in Figure 3), the mean genetic distances to their central nodes (0.76 and 0.44 substitutions with SEs of 0.09 and 0.12, respectively) give estimated ages of 8360 ± 900 years and 4840 ± 1320 years, respectively. Similarly, the estimated ages of three subclusters of Clade B are 5940 ± 900, 5500 ± 2420 and 4950 ± 1540 years, based on mean genetic distances to their central nodes of 0.54 ± 0.09, 0.50 ± 0.22 and 0.45 ± 0.14 substitutions, respectively. The estimated ages of Clades C, D, and E are 6710 ± 1430, 8470 ± 3080 and 2750 ± 1430 years, based on mean genetic distances to their central nodes of 0.61 ± 0.13, 0.77 ± 0.28 and 0.25 ± 0.13 substitutions, respectively. The estimated domestication timescales of all identified clades or subclades are much shorter when the mean mutation rate under the exponential growth model (19.3 % per million years) was adopted (see Additional file 3). When the mutation rate of bison (32 % per million years) according to the radiocarbon-dated sequences was adopted [30], the possible domestication and expansions in Clades A, B, C and D were dated between 1200 and 3700 yrs BP (see Additional file 3).

## Discussion

### Maternal lineages of the domestic yaks and their origins

Evidence of deep maternal diversity has been found in most domesticated ungulate animals [5-11,21,22]. Here we present evidence for similarity divergence in yaks – one important domesticated ungulate animal that had not previously been analyzed in this fashion. The results, together with those of the cited studies, suggest that deeply diverged lineages might be a general characteristic of most large animals.

We estimated, from our molecular calibration, that the two main lineages in domestic yaks probably diverged > 100,000 yr BP (see Additional file 2), far earlier than yaks could have been domesticated. Therefore, it is unlikely that the deep divergence resulted from two lines derived from different domestication events following separate evolutionary paths. This raises interesting questions about how such deep divergence may have arisen in the wild population. According to theoretical modelling, the deep maternal lineages detected may have originated either from distinct wild populations or from a single large population containing highly divergent lineages [5]. However, here we provide robust evidence showing that these two main lineages are also present in a single wild population, suggesting that their divergence occurred before the domestication. This phylogenetic pattern suggests that all domestic yaks may have originated from a single wild population (or gene pool). Notably, only one haplotype in the network was found within both wild and domestic yaks, indicating that the wild yaks sampled in this study are unlikely to be derived from domestic animals. Therefore, the first simple inference is that the size of the ancestral population was large enough to give rise to these two deep lineages. Assuming a 3-year generation length in yaks, a divergence time of > 100,000 yr BP suggests that more than 300,000 reproductive females would have been required for such deep divergence under the constant population size hypothesis. Both pastoralism and other human activities (such as intensive hunting) have greatly decreased the number of wild yaks during the past century [14,17]. However, it seems unlikely that a population of this size was present at the time because the number of wild yaks in the present QTP is only estimated to be ca. 15,000 individuals [14]. Another possible explanation for the occurrence of two such deeply diverged lineages is that the glacial climate drove the wild yaks into two refugia, where the populations diverged, then mixed again in interglacial or postglacial periods. This is highly possible since the estimated date of divergence (between 86,000 and 157,000 yr BP) falls well within the Quaternary. At this stage, the QTP was subjected to several cycles of glacial and interglacial climatic oscillation [31]. Geological evidence suggests that during the maximum glacial advance, an ice sheet covered an area five to seven times larger than it does today [32]. The pollen fossil records suggest that vegetation of the central QTP shifted alternately between permafrost-steppe and forest in response to the Quaternary glaciations and interglaciations, respectively [33]. The lack of food might have driven yaks into different adjacent refugia, and thus accumulate sufficient divergence to explain the divergence we observed. However, when the climatic oscillation ended, wild yaks might have returned to the high QTP and mixed because they could not endure the high temperature in the low-altitude regions. Further analyses of more wild samples and developments of new statistic models are needed to clarify which of these explanations is the most likely.

We initially expected the maternally identified clades to show a correspondence with the current breeds, because these breeds have distinct morphological differences [17]. However, the data we obtained show no distinct correlations (Table 3), suggesting that the morphological diversification might have occurred late after their maternal divergences, especially between the two deep lineages. Similar situations have also been found in other animals (such as chickens, [22]) and have been suggested to be due to extensive interchanges between different maternal lineages during trade migrations of human beings in early herding history. However, such a hypothesis relies on the assumption that the lineages derived from separate domestications of different wild populations separated by substantial geographical distances. For domestic yaks, the mixtures of maternal lineages found in most morphological breeds are mainly due to the common presence of both lineages in the wild populations and the limited distribution of wild yaks in the QTP. According to this scenario, any domestication event might have recruited wild individuals from both lineages. Furthermore, it is conceivable that the high mobility of nomadic pastoralists could have resulted in the mixture of the different lineages if they had been domesticated at different times or in different places in or close to the wild distribution. Furthermore, the divergence of all the identified clades within the two lineages seems to have been completed in the wild population before the domestication(s). The three clades that are represented in relatively small numbers of individuals (D, E and F) do not contain wild haplotypes, but all of the three main clades (A, B and C) do. The four former clades are situated at the base of the phylogenetic tree, suggesting that their haplotypes have earlier origins than those in the latter set of three clades. The failure to detect corresponding wild haplotypes in the four small groups (Clades D, E and F) is probably mainly due to the small number of wild yaks sampled. Therefore, the morphological diversification of current breeds probably occurred after stable pastoralist communities had been established. These findings are consistent with the recent hypothesis that the recent breeding events may have played a substantial role in the evolution of morphological breeds of domestic animals [34], probably due to a general relaxation of selective constraints in the wild animals [35].

**Table 3:** The distribution of sampled individuals of 10 morphological breeds in the identified seven phylogenetic clades (see Fig. 2).

Breed	Clade A	Clade B	Clade C	Clade D	Clade E	Clade F
Tianzhu	9	4		1	1	
Datong	17	7	4	2	1	
Huanhu	5	4				
Plateau	52	13	9	6	5	
Jiali	18	5	4	6	3	
Sibu	2				1	
Pali	14	4	4	3		
Maiwa	2		3			
Jiulong	4		1	1	1	
Bazhou	21	12				2

**Table 4:** Nucleotide diversity and Fu's *F*s values of the major clades in the sampled yaks

Clade	Including Wild yaks			Excluding Wild yaks		
		
	No	π	*Fs*^a^	No	π	*Fs*
A	159	0.00262 ± 0.00171	-27.888**	155	0.00258 ± 0.00169	-27.906**
B	56	0.00272 ± 0.00178	-16.429**	54	0.00249 ± 0.00167	-14.246**
C	33	0.00180 ± 0.00133	-9.005**	31	0.00163 ± 0.00124	-6.583**
D	18	0.00136 ± 0.00112	-2.916**	18	0.00136 ± 0.00112	-2.916**
E	12	0.00079 ± 0.00061	-2.124**	12	0.00079 ± 0.00061	-2.124**

^a^Fu's *F*s test (Fu, 1997); No, the number of the sampled individuals

**Significant Fu's *F*s values (*P *< 0.01).

### Domestication of yaks in the Qinghai-Tibetan Plateau

Populations near to a centre of initial origin are expected to maintain more ancestral variation and to show higher haplotypic and nucleotide diversity than populations derived through subsequent migration colonization [10]. However, recent gene flow after domestication might blur this genetic signature. The distribution of the seven domestic phylogenetic clades shows that the total haplotype diversity is similar among the geographical regions. This pattern might result from the frequent gene flow among breeds and different regions after the domestication. However, both the total number of haplotypes and the number of haplotypes unique to the specific region is high in Qinghai and Tibet, which are closer to the current wild distribution (Table 2, Fig. 1). After adjusting the frequencies to account for differences in sample sizes there were also significantly more haplotypes and endemic haplotypes in Qinghai and Tibet than in the other regions (*P *< 0.05). This high diversity is consistent with the previous suggestion by Qian [1] that the earliest domestication events occurred in this region. Further details regarding the probable evolution and spread of the haplotypes provided by the median networks also support this hypothesis (Fig. 2). The phylogenetic patterns indicate that haplotypes of Clades A, B and C found in Gansu, Sichuan, and Xinjiang derived from a subset of the Qinghai and Tibet haplotypes. The regionally restricted occurrence of Clades F and G may represent later introgressions of wild yak sequences into the domestic population. However, since these clades were found in only two and three individuals, respectively, it is possible that they were formed together with the other clades and are also widespread, but were not detected in the other regions because they occur at low frequency. These findings suggest that (a) domestication event(s) occurred at a place (or places) fairly close to the centre of the wild distribution and the initial mitochondrial gene pool then spread from the region of origin to the other regions, the unique types subsequently forming in the different derived domestic yak populations.

However, the available genetic data do not allow definitive conclusions to be drawn on whether yaks were domesticated in single or multiple events from the single wild gene pool. Under the hypothesis that all current domestic yaks might have originated from a single domestication, all clades should have similar genetic patterns and expansion times because their wild founders probably originated in the wild populations before their domestication. However, Clades A, B, C and D expanded earlier than the other clades according to our dating of the expansions of the presumed wild founders. Therefore, these demographic differences seem to support an alternative hypothesis that multiple domestications occurred from the same gene pool at different times. However, we were unable to discern how many wild haplotypes were actually involved in the founding of each clade due to the limited numbers of wild yak samples. The differences in founding haplotypes would undoubtedly have affected the network patterns of each clade and the consequent dating. For example, the greater age of the first subcluster (A1) in Clade A and its network pattern suggest that more than one haplotype might have founded this subclade, and further subdivision of A1 should reduce the current calibration (Fig. 3). This dilemma would presumably be resolved by the analysis of more wild and domestic samples.

Because of the great difference of the mutation rates between the shorter and longer time-scales, it is difficult to pinpoint the initial domestication time in animals [34]. This is probably due to the deleterious mutation accumulations and fewer purifying effects on the shorter time-scales [36,37]. We firstly calculated the domestication timescales under the molecular clock hypothesis (the substitution rate, 1 substitution per 40,000 years for the analyzed 637-bp region). Our initial calculations suggested an unrealistic early dating of the start of the domestication (between 10000 and 30000 yrs BP for five identified clades). Because of the lack of the archeological sequences in yaks, it is unlikely to obtain the actual pedigree mutation rate in this animal. The mutation rate of bison [30] is probably unsuitable for yaks, because the dated domestication time (1200–3700 yrs BP, see Additional file 3) is obviously shorter than the historical records of yak husbandry [38]). However, the timescales estimated according to the mutation rate calibrated between bison and yak divergence under the molecular clock relaxation allowing the terminal branches to have faster rates suggested that the domestication of yaks probably occurred around the early Holocene, within 10,000 yr BP [see Additional file 3] This hypothesized scenario is more consistent with the available archeological data for both colonization signatures of human beings in the QTP and yak remains. It remains unknown whether the earliest human being who colonized the QTP ca. 30,000 yrs BP had survived the Last Glacial Maximum between ca. 20,000 and 15,000 yrs BP [39]. The undoubted postglacial evidence of human colonization of the QTP was found in the north-eastern part, in what is now Qinghai, at least ca. 12,000 yrs BP [40] and in Tibet before 10,000 yrs BP [41]. These colonizers, possibly as well as earlier survived ones may have initiated the domestication of yaks. The yak remains found in Tibet, dating from ca. 10,000 yrs BP [1], might have suggested the earliest domestication of yak. The archaeological records further suggest that the abundance of yak products increased several-fold between 10,000 and 5,000 yrs BP, and by 4,500 yrs BP pastoralist communities were well established in the QTP [38]. These findings suggest that the yak husbandry might have grown rapidly after the initial domestication since 10,000 yrs BP. In addition, it is interesting that this growth is closely correlated with the rapid vegetation transition that occurred during the early Holocene in the eastern QTP. It remains unclear why the forests that recolonized this region postglacially were quickly replaced by steppe and alpine meadow vegetation [42], which has persisted to the present day. However, the development of yak husbandry after the initial domestication may be one of the factors that caused the forests to disappear and promoted the persistence of steppe and meadow vegetation through top-down controls of the alpine ecosystem.

## Conclusion

The genetic evidence presented in this study suggests that the deeply divergent maternal lineages characteristic of most domesticated ungulates are also present in domestic yaks. The presence of deeply diverged lineages of domestic individuals in a single, small wild population was first demonstrated in ungulate animals. This finding provides robust evidence that deeply diverged lineages of domestic animals may very well originate from a single gene pool, rather than from genetically discrete populations. Although our data do not allow definitive conclusions to be drawn on whether yaks were domesticated in single or multiple events, the molecular calibrations based on the demographic expansion suggest that domestication may have occurred within the Holocene, which is consistent with the archaeological records. This elucidation of the origin of domesticated yaks extends our broad understanding of the main ungulate domestications during the history of human civilization. The practice of animal domestication has long been believed to have first developed in the Near East [2]; however, available information suggests that the practice reappeared in diverse locations of the world. Both archaeological and molecular evidence suggest that modern dogs were domesticated from wolves in eastern Asia about 15,000 yrs BP [10], sheep and goats from wild progenitors in the Near and Middle East around 10,000 yrs BP [5,9], cattle from aurochs in the Near East about 10,000 yrs BP [6], domestic donkeys from two wild progenitor lineages in Africa 5000 yrs BP [11], modern pigs from more than one boar population in multiple places across Eurasia since the Holocene [7] and domesticated horses from similarly distinct horse populations across Eurasia since 4,500 yrs BP [8]. The cited reports, together with the present study suggest that domestication of local wild prey ungulate animals has been a common practice after the postglacial human colonization in widely varying locations of the world. The early humans tamed local populations of both widely distributed wild species (e.g. boar) and locally endemic species (e.g. yak). These early domestic animals then dispersed throughout larger areas, or even across the world, carried by humans during migrations or along trade routes. Despite the common general feature of deeply divergent maternal lineages, the demographic histories of these ungulate livestock species are more complex and variable than previously thought. For yaks, more wild samples are needed to clarify the development of the deeply divergent lineages and clades in the wild yaks, while more archaeological evidence will be helpful to elucidate the details of the early domestication and migration of yaks.

## Methods

### Samples

We examined DNA from 275 domestic animals representing 10 breeds of yak (Plateau, Huanhu, Da Tong, Tianzhu, Pali, Sibu, Jianli, Jiulong, Maiwa and Bazhou), including sequences of 25 yaks downloaded from GenBank. The breeds are classified by their morphological characteristics rather than their local origins. Therefore, the domestic yaks were subdivided into five geographical groupings: Qinghai, Tibet, Gansu, Sichuan and Xinjiang. The tissues of eight wild yaks were collected from one small population in west Qinghai, all of which died of unclear causes in March 29, 2005. Materials for another five wild yaks were collected from the Datong yak breeding facility (Qinghai) and the museum of the Northwest Plateau Institute of Biology, the Chinese Academy of Sciences, which were captured or killed on the QTP, but no precise details of the locations of the captures or kills are available (for further information see Table 1).

### Extraction, DNA amplification and sequencing

DNA was extracted from frozen muscle tissue, blood or dried skin using a standard phenol-chloroform method [8-10]. The mitochondrial D-loop region was amplified using the primers YDF 5'-GTAAAGAGCCTCACCAGTAT-3' and YDR 5'-TCCTGTAGCCATTGACTAT-3'. PCR amplifications were carried out in 30 μl reaction mixtures including each primer (0.3 μl of a 10 μM solution), dNTPs (0.7 μl of a 2.5 mM solution), 3 μl of 10 × buffer and 0.12 μl of 5 U/μl Taq DNA polymerase (Takara). The PCR program consisted of an initial denaturing step at 95°C for 4 min, 35 amplification cycles (95°C for 55 s, 50°C for 55 s and 72°C for 55 s) and a final extension at 72°C for 5 min in a Tpersonal Thermocycler (Biometra). PCR products were purified using a CASpure PCR Purification Kit following the manufacturer's recommended protocol (Casarray, Shanghai, China). Sequencing reactions were carried out using a DYEnamic Dye Terminator Cycle Sequencing Kit (Amersham Pharmacia Biotech Inc.), also following the manufacturer's recommended protocol. Sequencing products were separated and analyzed using a Megabase 500 Automated sequencer (Amersham Pharmacia Biotech Inc.).

### Analysis of mitochondrial sequence data

The sequences were manually aligned and new sequences were submitted to GenBank with accession numbers [GenBank: DQ138998 to DQ139260]. The 25 downloaded sequences are under the accession numbers [GenBank: AY521137–AY521161, AY722118 and AY749414]. One sequence of *Bison *(GenBank: U12936) was used as outgroup. At first, we constructed a neighbor-joining (NJ) tree of all the haplotypes under the Kimura 2-parameter model using MEGA 3 software [43]. Then, phylogenetic relationships of haplotypes were further confirmed by maximum parsimony and maximum likelihood analyses in PAUP* 4.0b10 [44] and Bayesian analyses [45]. Maximum parsimony analyses (equally weighted characters and nucleotide transformations) involved a heuristic search strategy with 100 replicates of random addition of sequences, in combination with ACCTRAN character optimization and MULPARS+TBR branch-swapping and STEEPEST DESCENT options on. The program Modeltest [46], was utilized to find the model of sequence evolution that best fit data set by the hierarchical likelihood ratio (LR) test for maximum likelihood analyses. Maximum likelihood heuristic search parameters were simple addition sequence of taxa with TBR branch swapping, MULTREES and COLLAPSE. For Bayesian analyses [45], four simultaneous Monte Carlo Markov Chains (MCMC) were run for 5,000,000 generations, saving a tree every 1000 generations. Posterior probability (shown as percentages, PP) for Bayesian analyses [45] and bootstrap values (BP) [47] for MP trees assessed relative support for monophyletic groups. Burn-in, the generation time for each parameter to reach the stationary state, was determined by visual inspection of the log-likelihood values. We discarded the first 499 trees and collected 4501 trees (whose log-likelihoods converged to stable values) to construct a 50% majority rule consensus tree with posterior probabilities with PAUP* v4.0b10 [44]. Bootstrap values were calculated from 1000 replicates using a heuristic search with simple addition with TBR and MULPARS options on (maximum parsimony) and a fast stepwise-addition search of the maximum-likelihood genetic values obtained (maximum likelihood).

At last, in order to investigate the possible relationships among all sequences of each major clade identified in the above analyses, median-joining networks were constructed using the program Network 4.1 [48]. Arlequin 2.0 was used to estimate nucleotide diversity values, parameters and goodness of fit from mismatch distributions and to compute Fu's Fs test of selective neutrality values [49].

### Molecular calibration

In order to calculate the mutation rate for the studied region of the D-loop, we extended to compile a separate data matrix comprising all yak halotypes, seven sequences (U12864, U12936, U12946, U12948, U12955, U12956 and U12959) of *Bison bison *(the sister group of yak, [19]) and one sequence of *Bos incus *(L27714, outgroup). This matrix under the selected best-fit model of DNA substitutions [46] was subjected to maximum likelihood analyses for molecular clock hypothesis tests. The hypothesis of rate constancy was evaluated with the LR test by calculating the log likelihood score of the chosen model with the molecular clock enforced and comparing it with the log likelihood score without the molecular clock enforced. The molecular clock was rejected because constrained and unconstrained analyses differed significantly (HKY+I+G with a 15.93 ratio for transition/transversion, -2 ln LR = 120.94, df = 94, *P *= 0.03). We therefore used Bayesian analysis to estimate coalescent time of two deep lineages and evolutionary rate, as implemented by the program BEAST [50,51]. The molecular clock assumption was relaxed by allowing the rate to vary throughout the tree in an autocorrelated manner, with the rate in each branch being drawn from an exponential distribution whose mean was equal to the rate in its parent branch. Two population models (constant size and exponential growth) were tested, and the final rate estimates from the two models were compared. The mean mutation rate of the model that yielded the highest posterior probability was chosen. Following a burn-in of 5,00,000 cycles, all parameters were sampled once every 100 generation from 5,000,000 Markov Chain Monte Carlo (MCMC) steps. Convergence of the chains to the stationary distribution was checked by visual inspection of plotted posterior estimates using the program TRACER [52], and the effective sample size for each parameter sampled from the MCMC analysis was almost always found to exceed 100, usually by an order of magnitude. The divergence between yak and *Bison *was assumed to start around 1.8 myr ago, based on the fossil records and calibrations from the other molecular sequences [18,19]. Thus we calibrated the phylogeny with a normal distribution prior (mean: 1.8 myr, std: 0.2 myr) on the divergence time of *Bison *and yak. Then we used our previous method to calculate the probable domestication time during the domestication expansion of each identified clade according to the estimated rate [10]. Because the evolutionary rate is dependent on the calibration point, mutation rates observed in pedigrees are higher than those inferred from the earlier species divergence times [34-37]. For a comparison, we further used the mean mutation rate of bison, 3.2 × 10^-7 ^(substitution/site/year), based on the radiocarbon-dated sequences [30] to estimate the possible domestication expansion timescales in each identified clade.

## Authors' contributions

JQL and XQZ conceived and designed research; SCG, JPS, QZ and DLQ collected materials and performed experiments; JQL, SCG, JZ and YZ analyzed data, and JQL, PS and SCG wrote the paper.

## Supplementary Material

Additional File 1 The 50 % majority-rule consensus tree of 10000 trees with 159 steps, a consistency index (CI) of 0.560, and a retention index (RI) of 0.895. These trees were produced by the maximum parsimony analyses in PAUP* 4.0b10. Click here for file

Additional File 2 The estimated coalescent time of two deep divergent lineages. These timescales were calculated through Bayesian analyses assuming the constant size and exponential growth model based on the calibration point between yak and bison (1.8 myr) Click here for file

Additional File 3 The estimated domestication time in five major clades. These timescales were calculated according to the three assumed different mutation rates. Click here for file
