# Supplementary figures and images for: Origin of mitochondrial DNA diversity of domestic yaks
Source: BMC Evol Biol. 2006 Sep 22;6:73. doi: 10.1186/1471-2148-6-73 (PMC1626082; doi:10.1186/1471-2148-6-73)

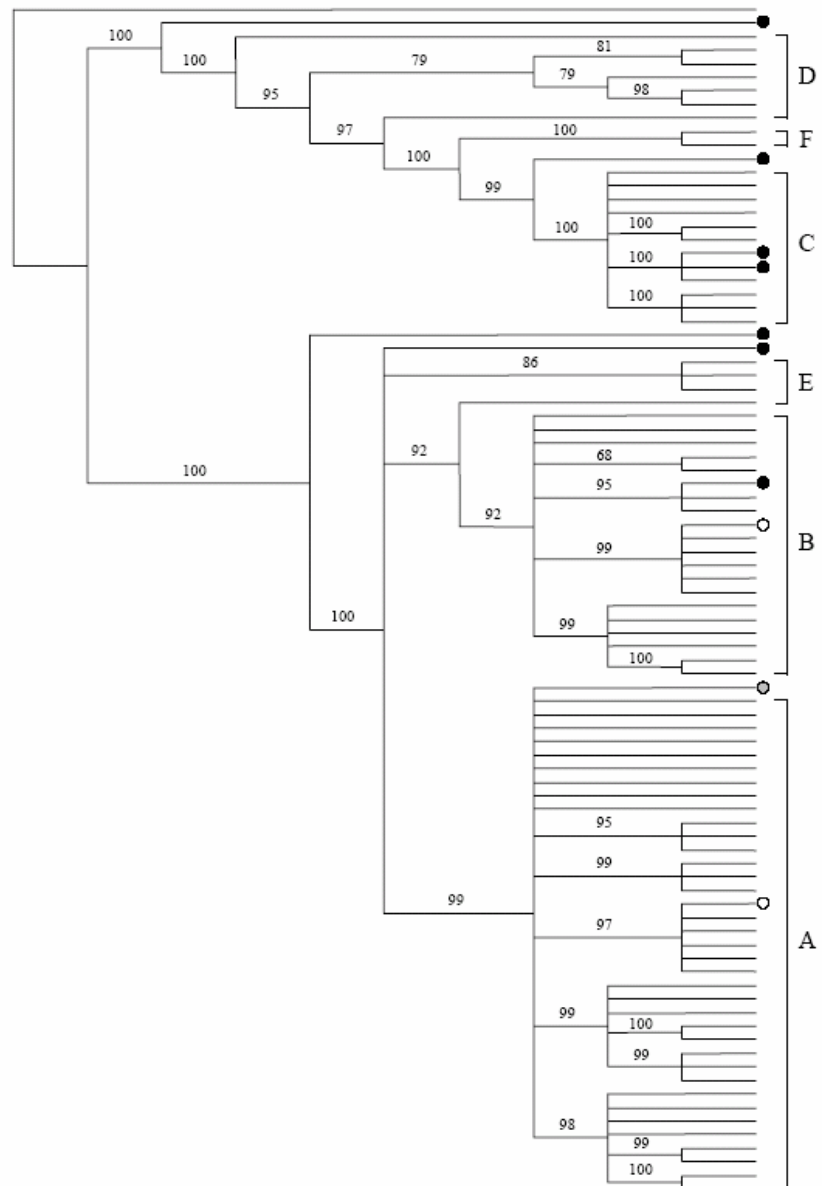

Supplement: Additional File 1 — The 50 % majority-rule consensus tree of 10000 trees with 159 steps, a consistency index (CI) of 0.560, and a retention index (RI) of 0.895. These trees were produced by the maximum parsimony analyses in PAUP* 4.0b10. [file 1471-2148-6-73-S1.pdf]
