# Supplementary material for: Origin of mitochondrial DNA diversity of domestic yaks
Source: BMC Evol Biol. 2006 Sep 22;6:73. doi: 10.1186/1471-2148-6-73 (PMC1626082; doi:10.1186/1471-2148-6-73)
Supplement: Additional File 2 — The estimated coalescent time of two deep divergent lineages. These timescales were calculated through Bayesian analyses assuming the constant size and exponential growth model based on the calibration point between yak and bison (1.8 myr) [file 1471-2148-6-73-S2.doc]

|  | Constant size  Lower Mean Upper | | | Exponential growth  Lower Mean Upper | | |
| --- | --- | --- | --- | --- | --- | --- |
| Root height (kyr B.P.) | 104.46 | 131.38 | 157.45 | 86.77 | 109.36 | 131.59 |
| Ln (posterior) | -1853.44 | -1889.38 | -1927.60 | -1899.76 | -1862.21 | -1826.97 |
| Sitemodel alpha | 0.18 | 0.69 | 1.38 | 0.19 | 0.61 | 1.3 |
| Sitemodel pInv | 0.60 | 0.72 | 0.83 | 0.58 | 0.70 | 0.80 |
| Mutation rate (% per Myr) | 9.1 | 14.6 | 21.0 | 11.9 | 19.3 | 28.2 |

Additional file 2

The estimated coalescent time of two deep divergent lineages and results of Bayesian analyses assuming the constant size and exponential growth model based on the calibration point between yak and bison (1.8 Myr).
