# Supplementary material for: Origin of mitochondrial DNA diversity of domestic yaks
Source: BMC Evol Biol. 2006 Sep 22;6:73. doi: 10.1186/1471-2148-6-73 (PMC1626082; doi:10.1186/1471-2148-6-73)
Supplement: Additional File 3 — The estimated domestication time in five major clades. These timescales were calculated according to the three assumed different mutation rates. [file 1471-2148-6-73-S3.doc]

Additional file 3

The estimated domestication timescales of five major clades (Fig. 3) according to the three different mutation rates

| Subclades  /clade (Fig. 3) | Mutational distance to central node (mean ± S.E.) | Mean mutation rate (% per Myr) | | |
| --- | --- | --- | --- | --- |
|  |  | 14.6 (Constant size) | 19.3 (Exponential growth) | 32 [bison, 30] |
| A1 | 0.76 ± 0.09 | 8360 ± 900 years | 6080 ± 720 years | 3728 ± 441 years |
| A2 | 0.44 ± 0.12 | 4840 ± 1320 years | 3930 ± 960 years | 2158 ± 589 years |
| B1 | 0.54 ± 0.09 | 5940 ± 900 years | 4320 ± 720 years | 2649 ± 441 years |
| B2 | 0.50 ± 0.22 | 5500 ± 2420 years | 4000 ± 1760 years | 2453 ± 1079 years |
| B3 | 0.45 ± 0.14 | 4950 ± 1540 years | 3600 ±1120 years | 2207 ± 687 years |
| C | 0.61 ± 0.13 | 6710 ± 1430 years | 4880 ± 1040 years | 2992 ± 638 years |
| D | 0.44 ± 0.18 | 8470 ± 3080 years | 3920 ± 1440 years | 2158 ± 883 years |
| E | 0.25 ± 0.13 | 2750 ± 1430 years | 2000 ± 1040 years | 1226 ± 638 years |
